# Evaluation of the Antifungal Activity of *Bacillus* *amyloliquefaciens* and *B. velezensis* and Characterization of the Bioactive Secondary Metabolites Produced against Plant Pathogenic Fungi

**DOI:** 10.3390/biology11101390

**Published:** 2022-09-23

**Authors:** Shereen A. Soliman, Mona M. Khaleil, Rabab A. Metwally

**Affiliations:** 1 Botany and Microbiology Department, Faculty of Science, Zagazig University, Zagazig 44519, Egypt; 2 Biology Department, Faculty of Science, Taibah University, Yanbu El-Bahr 46429, Saudi Arabia

**Keywords:** antifungal activity, *Bacillus amyloliquefaciens*, endophytic bacteria, plant pathogenic fungi, secondary metabolites

## Abstract

**Simple Summary:**

Plant pathogenic fungi cause serious damage in agriculture, resulting in major losses in the yield and the quality of different economic crops. Chemical fungicides are dangerous to human health and the environment. They have many harmful side effects on non-target organisms. Moreover, their residues have been found in human food. Endophytic bacteria could be a valuable and safe alternative method for the biological control of phytopathogenic fungus. The endophyte *Bacillus amyloliquefaciens* provides a strong prospect as a biocontrol agent against *Alternaria* sp. on pepper plants.

**Abstract:**

Endophytic bacteria are plant-beneficial bacteria with a broad host range. They provide numerous benefits to their hosts, helping them tolerate several biotic and abiotic stresses. An interest has recently been developed in endophytic bacteria which are producing bioactive compounds that contribute to the biological control of various phytopathogens. This research paper aimed to investigate the potentiality of new local strains of endophytic bacteria such as *Bacillus amyloliquefaciens* and *B. velezensis* and the production of several antimicrobial metabolites associated with the biocontrol of *Alternaria* sp., which cause serious diseases and affect important vegetable crops in Egypt. Twenty-five endophytic bacteria isolates were obtained from different plants cultivated in El-Sharkia Governorate, Egypt. Dual culture technique was used to evaluate the bacterial isolates’ antagonistic potentiality against *Alternaria* sp. and *Helminthosporium* sp. The most active bacterial isolates obtained were selected for further screening. The antifungal activity of the most active endophytic bacterial isolate was assessed in vivo on pepper seedlings as a biocontrol agent against *Alternaria* sp. A significant antifungal activity was recorded with isolates C_1_ and T_5_ against *Alternaria* sp. and *Helminthosporium* sp. The bacterial endophyte discs of C_1_ and T_5_ showed the highest inhibitory effect against *Alternaria* sp. at 4.7 and 3.1 cm, respectively, and *Helminthosporium* sp. at 3.9 and 4.0 cm, respectively. The most active endophytic isolates C_1_ and T_5_ were identified and the 16S rRNA sequence was submitted to the NCBI GenBank database with accession numbers: MZ945930 and MZ945929 for *Bacillus amyloliquefaciens* and *Bacillus velezensis*, respectively. The deformity of pathogenic fungal mycelia of *Alternaria* sp. and *Helminthosporium* sp. was studied under the biotic stress of bacteria. The culture filtrates of *B. amyloliquefaciens* and *B. velezensis* were extracted with different solvents, and the results indicated that hexane was the most efficient. Gas Chromatography-Mass Spectrometry revealed that Bis (2-ethylhexyl) phthalate, Bis (2-ethylhexyl) ester, and *N*,*N*-Dimethyldodecylamine were major constituents of the endophytic crude extracts obtained from *B. amyloliquefaciens* and *B. velezensis*. The in vivo results showed that *Alternaria* sp. infection caused the highest disease incidence, leading to a high reduction in plant height and in the fresh and dry weights of pepper plants. With *B. amyloliquefaciens* application, DI significantly diminished compared to *Alternaria* sp. infected pepper plants, resulting in an increase in their morphological parameters. Our findings allow for a reduction of chemical pesticide use and the control of some important plant diseases.

## 1. Introduction

Fungal diseases are a massive threat to crop yields and global food security. They destroy one-third of all food crops each year, causing economic losses and affecting global poverty levels [1,2]. Many of these fungi survive in the soil for extended periods as resting structures. Chemical fungicides have been used to control and avoid pathogenic fungi. However, in addition to the high cost, using these chemical compounds has limited efficacy and a considerable negative influence on non-target species and human health in the environment [3]. The development of safe alternatives to traditional fungicides has been prompted by researchers who are concerned about their effects on the environment and humans.

The biological methods of controlling diseases that affect plants is an eco-friendly alternative, especially applicable when there is pesticide resistance and the need for environmental protection toward sustainable agricultural methods [4,5]. Microbial antagonists have unique properties that inhibit fungal infection growth through direct and indirect processes [6,7]. The direct impact is mainly owing to the biocontrol agent’s antagonistic behavior against the pathogen due to competition, parasitism, antibiosis, and the production of extracellular digestive enzymes. In contrast, plant defense mechanisms are triggered in response to various pests and diseases, which has an indirect effect [7,8,9].

In pathological research, an endophytic bacterium suppressing plant diseases has received much attention [10]. Endophytes spend at least a part of their life cycle within the host plant and form a symbiotic relationship with it, making them highly effective biocontrol agents [11]. Endophytes live and survive within the stems, roots, and leaves of plants without causing disease symptoms [12,13]. Endophytic bacteria and their associations with their hosts have been studied to determine their ecological functions and assess their biotechnology potential [12]. Endophytes have been found to be an essential tool for improving crop performance compared to other biological agents since they colonize host tissue [14]. They can compete for nutrients as a colonizer of the roots and compete for space for their proliferation, resulting in the inhibition of pathogens. Furthermore, they do not pollute the environment [15].

Endophytic bacteria can be isolated from almost all plant species [12,16]. Plants’ pathogenic fungi, such as *Fusarium oxysporum*, *Sclerotium rolfsii* and *Rhizoctonia solani*, are inhibited by endophytic bacteria [17]. Amaresan et al. [16] found that endophytic bacteria that were isolated from *Capsicum annuum* have an antagonistic effect against several phytopathogens. Also, infection by fungal pathogens results in stronger reductions in plant biomass and survival compared to uninfected plants [12,18,19]. *Bacillus* was mentioned in numerous research papers due to its prevalence in many plants, its antibacterial properties [20,21,22], and its ability to produce endospores that are UV, pH, temperature, and salinity resistant [9]. This genus has become an attractive agent for commercial use in modern farming systems [13,23,24,25]. As a result, the current study aimed to screen and analyze new local strains of endophytic bacteria isolates for their ability to inhibit various phytopathogenic fungi, which cause serious diseases affecting important vegetable crops in Egypt, and this supports a reduction of chemical pesticides’ use. Furthermore, the antifungal activity of the most active endophytic bacterial isolate was assessed in vivo on pepper (*Capsicum annuum*) as a biocontrol agent against *Alternaria* sp.

## 2. Materials and Methods

### 2.1. Plant Materials Collection

Several crop plants were randomly collected from various locations in Minia Al-Qamh soils, El-Sharkia Governorate, Egypt, including leaves, roots, and stems from *Solanum melongena*, *S. lycopersicum*, *Allium cepa*, *Coriandrum sativum*, *Pisum sativum*, *Portulaca oleracea*, and *Brassica oleracea* (Table 1), and placed in polypropylene bags for transport to the laboratory.

### 2.2. Plant Segments Sterilization and Endophytes Isolation

Endophytes were isolated from different plant parts using the [26] method. Healthy samples of different plants were used. Segments were cut from the stems (1 cm), leaves (1.5 × 1 cm), and roots (1 cm) of each plant. They were washed in running water, sterilized for 10 min with sodium hypochlorite (NaOCl, 5%), rinsed three times with sterile distilled water, and dried on a sterilized filter paper. Sterilized segments were put into a nine cm Petri plate containing potato dextrose agar (PDA) medium and incubated at 30 °C. After two days, bacterial colonies were picked out [27] and were checked by successive subcultures on the agar medium. The purified bacteria were then stored at −20 °C in a nutrient broth of 20% glycerol.

### 2.3. Isolation of Pathogenic Fungi

Pathogenic fungi were isolated and randomly selected from symptomatic leaves collected from El-Sharkia Governorate, Egypt. The leaves were rinsed with tap water before being soaked in 5% sodium hypochlorite (NaOCl) for 10 min, rinsed three times in sterile distilled water, and dried on sterile filter paper. Sterilized segments were placed on a 9 cm Petri dish containing potato dextrose (PDA) medium with rose bengal 30 mg/L and 250 mg/L streptomycin. Plates were incubated for 10 days at 30 °C. Fungal colonies were purified [28] and identified according to culture and microscopic characteristics [29].

### 2.4. Primary Screening of the Antagonist Activity of Bacterial Isolates in Vitro

The endophytic bacterial isolates were tested for antagonistic activity against *Alternaria* sp. and *Helminthosporium* sp. using a dual culture technique. A ten-millimeter disc of a seven-day fungal pathogen culture was inoculated in the middle of Petri plates. The bacterial endophyte was streaked on the opposite side of the agar (PDA) plates. The plates inoculated with the pathogenic fungal disc were considered as the controls [30], and the plates were incubated at 28 ± 2 °C. The inhibition zone indicated the antagonistic properties of endophytic bacteria after seven days. Four replicates were measured for each isolate, and the experiment was performed twice to ensure accuracy. The following formula was used to calculate the percentage of radial growth inhibition relative to the control [31].

Percent of Inhibition % (I) = C − T/C × 100

where C-radial growth is the control and T-radial growth is the treatment.

The width of the inhibition zone was evaluated as + for 2–5 mm; ++ for 5–10 mm; and +++ for > 10 mm [32].

### 2.5. Evaluation of the Antifungal Activity of Endophytic Bacteria

PDA (20 mL) was inoculated with one mL of fungal spore suspension of *Alternaria* sp. and *Helminthosporium* sp. separately poured into a Petri dish of 90 mm in diameter. The plates were allowed to solidify and were then seeded with: (i) cell-free culture (150 µL) obtained by the cultivation of bacterial isolates in nutrient broth for 48 h and 150 rpm, and a millipore filter (0.45 μm) was used to filter-sterilize the culture supernatant using the well-diffusion method [33]; and (ii) bacterial discs (15 mm) from the edge of the active growing cultures of seven endophytic bacterial isolates at 48 h of age each. The plates were then left for 2 h in a refrigerator, after which they were incubated for five days at 28 °C. The inhibition zones were measured at the end of the incubation period. The most bioactive endophytic bacteria were selected for further investigations.

### 2.6. Morphological and Biochemical Characteristics of the Antagonistic Bacteria

The antagonistic bacteria were grown on nutrient agar for 24 h. The Gram stain technique was determined according to standard microbiological procedures [34]. Bergey’s Manual of Determinative Bacteriology was used to determine bacterial isolates’ physiological and biochemical features [35].

### 2.7. Molecular Characterization of Bacterial Isolates by Partial Sequencing of 16S rDNA

The DNA of the most active isolates was extracted using standard bacterial procedures [36]. PCR was used to preferentially amplify the 16S rRNA gene from genomic DNA using the universal forward primer (F1) 5′ AGAGTTTGATCCTGGCTCAG 3′ and the reverse primer (R1) 5′ GGTTACCTTGTTAC GACTT 3′ according to [37]. The 16S rRNA gene of the bacterial isolates was aligned with the standard reference sequences obtained from GenBank, NCBI, using BLAST (http://blast.ncbi.nlm.nih.gov/) (accessed on 1 September 2021).

A phylogenetic tree was created using MEGA 6.0 [38]. The sequence was finally submitted to GenBank, and an accession number was obtained.

### 2.8. Morphological Abnormalities in the Alternaria sp. and Helminthosporium sp. Hyphae due to the Antagonistic Effects of Endophytic Bacterial

The morphological deformation caused by the most bioactive endophytic bacterial strains on the mycelia of each pathogenic fungus (*Alternaria* sp. and *Helminthosporium* sp.) on PDA plates was examined. The hyphal strands from the confrontation lines at the end of the fungal colony were extracted and compared to the control plates under a light microscope (Leitz WETZLAR, Wetzlar, Germany) for anomalies [39].

### 2.9. Preparation of Antifungal Bacterial Crude Extracts using Different Solvents

The two selected endophytic bacterial strains were cultured by placing agar blocks of actively growing pure culture (10 mm in diameter) in three Erlenmeyer flasks (1 L), each containing 300 mL of sterile nutrient broth for each endophytic bacterial strain, and incubated at 32 ± 2 °C for 24 h with continuous shaking at 150 rpm/min. After the incubation period, stationary growth cultures were centrifuged at 4000× *g* for 30 min at 4 °C. Each bacterial strain’s cell-free filtrates were extracted with an equal volume of ethyl acetate, chloroform: methanol (2:1 *v*/*v*), and hexane separately in a separating funnel by shaking vigorously for 15 min. The mixtures were allowed to settle until two different layers appeared: the upper solvent and the lower aqueous layer. The extraction was repeated three times [40]. The crude extracts were tested for their biological activity (100 µL) using the filter paper diffusion method against both. *Alternaria* sp. and *Helminthosporium* sp. were put separately on PDA media and the solvents were used as a control. Gas Chromatography and Mass Spectrometry (GC-MS) were used to analyze the crude extracts.

### 2.10. Gas Chromatography and Mass Spectrometry (GC–MS)

The bioactive components in the crude extracts of two endophytic bacterial strains were identified by GC-MS [41]. The crude extracts were analyzed by GC–MS using a (Thermo Scientific TRACE 1310 Milan, Italy) Gas Chromatograph attached with an ISQ LT single quadrupole Mass Spectrometer detector fitted with DB5-MS, 30 m and 0.25 mm ID (J&W Scientific) in the Al-Azhar University’s Regional Center for Mycology and Biotechnology, Cairo, Egypt. The instrument’s temperature was initially set to 40 °C and sustained for 3 min. The temperature was raised to 280 °C at a rate of 5 °C/min at the end of this period and maintained for 5 min. Then, it was increased to 290 °C at a rate of 7.5 °C/min and kept for 1 min. The injection port temperature was kept at 200 °C, while the helium flow rate was held for 1 mL/min. An ionization voltage of 70 eV was used. The mass spectra of the extracts were compared to data from WILEY and NIST to identify the bioactive chemicals present in MASS SPECTRAL DATABASE libraries [42].

### 2.11. In Vivo Evaluation of B. amyloliquefaciens Effects against Alternaria sp.-Infected Pepper Plants under Greenhouse Conditions

The antifungal activity of *B. amyloliquefaciens* against *Alternaria* sp. was evaluated in a pot experiment in a greenhouse of the Botany and Microbiology Department, Faculty of Science, Zagazig University, with a temperature range of 23–30 °C and a relative humidity of 60–85% in a completely randomized design.

#### 2.11.1. B. amyloliquefaciens Inoculum Preparation

On a rotary shaker (180 rpm), *B. amyloliquefaciens* was grown at 30 °C for 48 h in nutrient broth. The bacterial suspension was obtained containing 10^7^ CFU/mL.

#### 2.11.2. Pot Experiment for Cultivation of Pepper Seedlings

Plastic bags (12 cm diameter and 22 cm high) containing sterile field clay soil (2 kg/ag) were collected from an agricultural field in Minia Al-Qamh, El-Sharkia Governorate. Experimental treatments were applied to 45–50 days old pepper seedlings procured from the local market. Initially, bacterial suspension, according to Widnyana and Javandira [43], was used to soak the roots of pepper seedlings for 4 h before transplantation; one seedling was transplanted per bag. Soils were drenched with 300 mL of the prepared inoculum or equivalent tap water. Two days after transplanting, the inoculation with the fungal pathogen *Alternaria* sp. was conducted by pipetting individual droplets of fungal suspension (10^5^ cfu/mL) on the surface of healthy leaves after gently removing the leaf wax of control and infected pepper leaves using a brush. Also, pepper plants pipetted with tap water droplets were used as control plants. After pathogen inoculation, the inoculated plants were kept under polyethylene bags for 24 h to ensure the infection process and maintain high humidity conditions. Then, they were exposed to greenhouse conditions. There were ten replicates (*n* = 10) for each particular treatment. Four weeks after inoculation, disease symptoms were recorded.

#### 2.11.3. Determination of Morphological Parameters

After four weeks of *B. amyloliquefaciens* application, pepper plants from the *Alternaria* sp. infected and non-infected treatments were uprooted and washed with tap water. The total heights of the pepper plants were measured. The total fresh weights (TFW) of the pepper plants for each treatment were taken, and then placed in the oven at 70 °C for two days. Their total dry weight (TDW) was recorded.

#### 2.11.4. Assessment of Disease Incidence (DI)

The incidence of the disease for the pepper plants that were only infected with *Alternaria* sp. and for the *Alternaria* sp. pepper plants infected and treated with *B. amyloliquefaciens*, was determined by the following formula:

$$\mathrm{Disease}\;\mathrm{Incidence}\;\left(\mathrm{DI}\right)\;\left(\%\right)=\frac{\mathrm{Number}\;\mathrm{of}\;\mathrm{infected}\;\mathrm{plants}}{\mathrm{Total}\;\mathrm{number}\;\mathrm{of}\;\mathrm{plants}}\times 100\;$$

### 2.12. Statistical Analysis

One-way ANOVA was used to analyze the data. To compare the means of the treatments, Duncan’s multiple range test at *p* < 0.05 was used. The software package statistics 10.1 was utilized for statistical analysis.

## 3. Results

**Figure 1:**
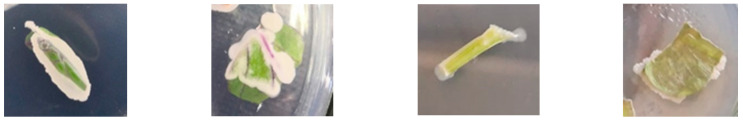
Endophytic bacteria isolated from different plant samples.

**Figure 2:**
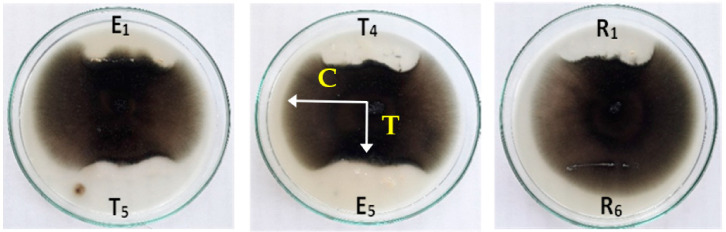
Dual culture plate method showing inhibition of radial growth of *Helminthosporium* sp. by different isolates of endophytic bacteria, where **C** is the growth of fungus toward the control side of the Petri dish and **T** is the outward growth of the fungus in the direction of the antagonistic bacteria.

In the present study, twenty-five isolates of endophytic bacteria were isolated from different plants collected from El-Sharkia Governorate, Egypt, as recorded in (Table 1) and shown in (Figure 1). From the leaves, stem, and roots of *Solanum melongena*, five isolates (E1, E2, E3, E4, and E5) were identified, while four isolates (T_1_, T_2_, T_4_, and T_5_) were isolated from *S. lycopersicum* leaves. On the other hand, only one isolate (C_1_) was isolated from *Brassica oleracea*. These isolates were examined to see whether they had high anti-*Alternaria* sp. and anti-*Helminthosporium* sp. effects using the dual culture technique (Figure 2). Compared to the control, the inhibitory zone of radial growth revealed endophytic bacteria’s antagonistic activity. The width of the inhibition zone between the pathogen and the antagonist was calculated as: + for 2–5 mm; ++ for 5–10 mm; and +++ for > 10 mm. The most active isolates were T_5_ and C_1_ against both *Alternaria* sp. and *Helminthosporium* sp. As shown in Table 1, R1 and R_2_ had moderate inhibition zones with both pathogens. In contrast, R_3_ and R_6_ did not have any inhibitory effects on either of the fungal pathogens.

### 3.1. Antifungal Activity of Cell-Free Culture and Discs of Endophytic Bacteria

**Figure 3:**
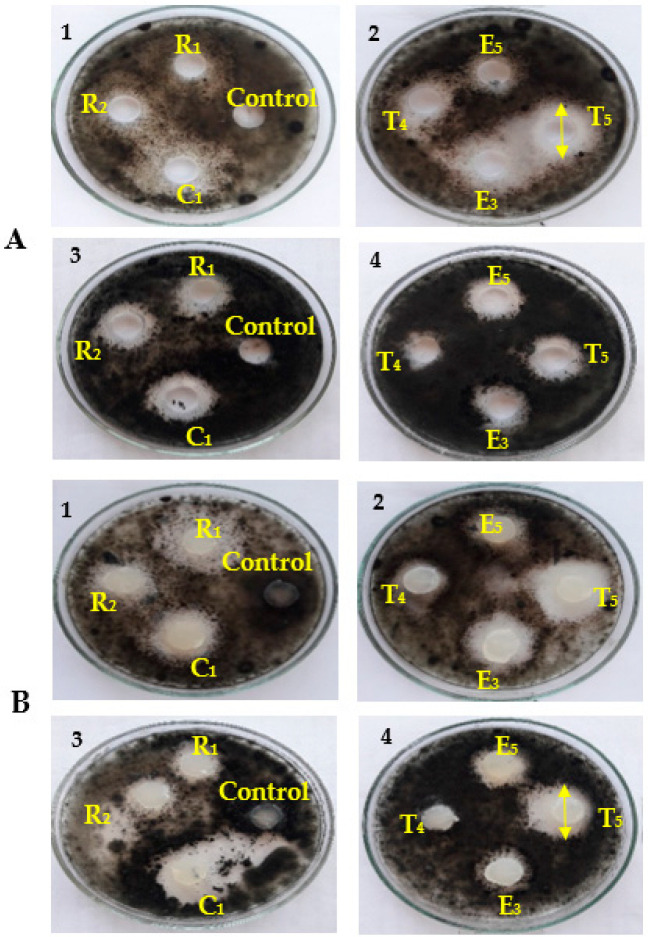
Inhibitory effects of selected endophytic bacterial isolates (E_3_, E_5_, R_1_, R_2_, T_4_, T_5,_ and C_1_) against *Alternaria* sp. and *Helminthosporium* sp. using the well diffusion method (**A**) and the disc diffusion method (**B**).

The results in Table 2 and Figure 3 indicate that the cell-free culture was effective (150 μL) or that the discs (15 mm) of endophytic bacteria isolated from *P. oleracea* (isolates R_1_ and R_2_), *S. lycopersicum* (isolates T_4_ and T_5_), *S. melongena* (isolates E_3_ and E_5_), and *B. oleracea* (isolate C_1_) had exhibited inhibitory activities against *Alternaria* sp. and *Helminthosporium* sp. Moreover, the bacterial endophyte discs of C_1_ and T_5_ isolated from the leaves of *B. oleracea* and *S. lycopersicum*, respectively, showed the highest inhibitory effects against *Alternaria* sp. (4.7 ± 0.252 and 3.1 ± 0.164 cm, respectively) and *Helminthosporium* sp. (3.9 ± 0.329 and 4.0 ± 0.212 cm, respectively) as compared to the other isolates.

### 3.2. Identification of Endophytic Bacterial Isolates

The morphological and biochemical characteristics of the antagonistic bacteria are listed in Table 3. Positive biochemical results involve catalase, oxidase, and the hydrolysis of gelatin and starch. Negative results include the indole test, hydrogen sulfide, the methyl red test, and urease.

**Figure 4:**
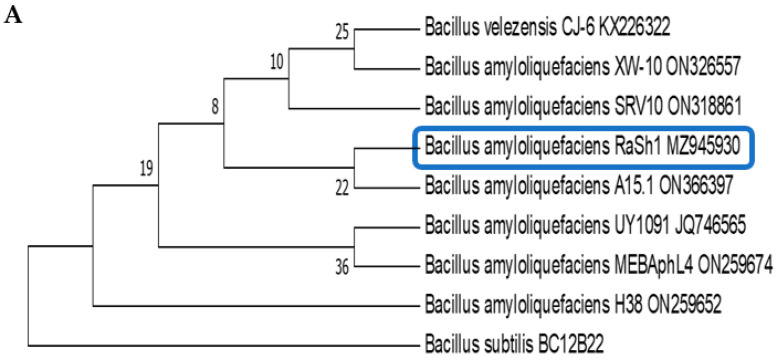

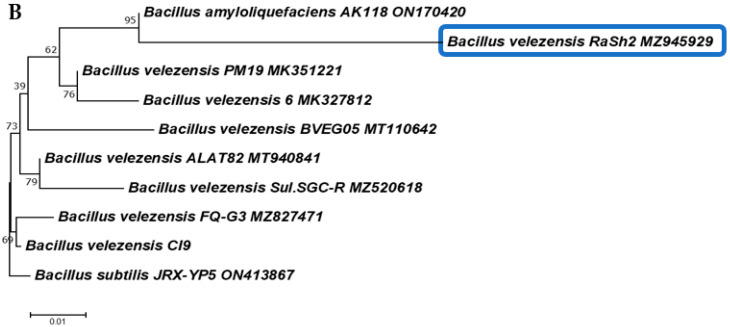
Phylogenetic analysis of *B. amyloliquefaciens* RaSh1 (MZ945930) (**A**), and *B. velezensis* RaSh2 (MZ945929) (**B**), showing their relationship with the ITS sequences of closely related Bacillus strains retrieved from the NCBI GenBank database. The percentage of replicate trees in which the associated taxa clustered together in the bootstrap test (1000 replicates) is shown next to the respective branches. The evolutionary distances were computed using the Maximum Composite Likelihood method and are in the units of the number of base substitutions per site. Evolutionary analyses were conducted in MEGA7 program. *Bacillus subtilis* BC12B22 is used as an outgroup for *B. amyloliquefaciens* RaSh1, and *Bacillus subtilis* JRX-YP5 ON413867 is used as an outgroup for *B. velezensis* RaSh2.

The most active endophytic bacterial isolates were selected and verified using the 16S rDNA gene sequence. The obtained partial sequence of the 16S rDNA gene was deposited in the GenBank database under accession numbers; MZ945930 and MZ945929, respectively, as shown in Figure 4.

### 3.3. Morphological Changes under the Light Microscope

**Figure 5:**
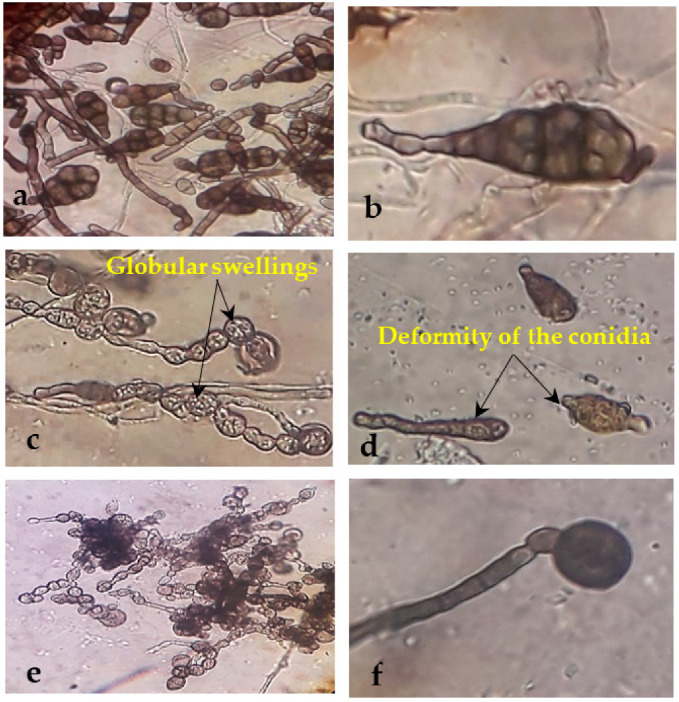
Morphological abnormalities in the mycelia of *Alternaria* sp. upon interaction with endophytic bacteria. Images (**a**,**b**) show the untreated (control) *Alternaria* sp. mycelia and spores. (**c**,**d**) show the swelling and deformity of *Alternaria* sp. mycelia and spores treated with *B. velezensis*. (**e**,**f**) were representatives of the segmentation and deformation of *Alternaria* sp. mycelia and spores treated with *B. amyloliquefaciens*.

**Figure 6:**
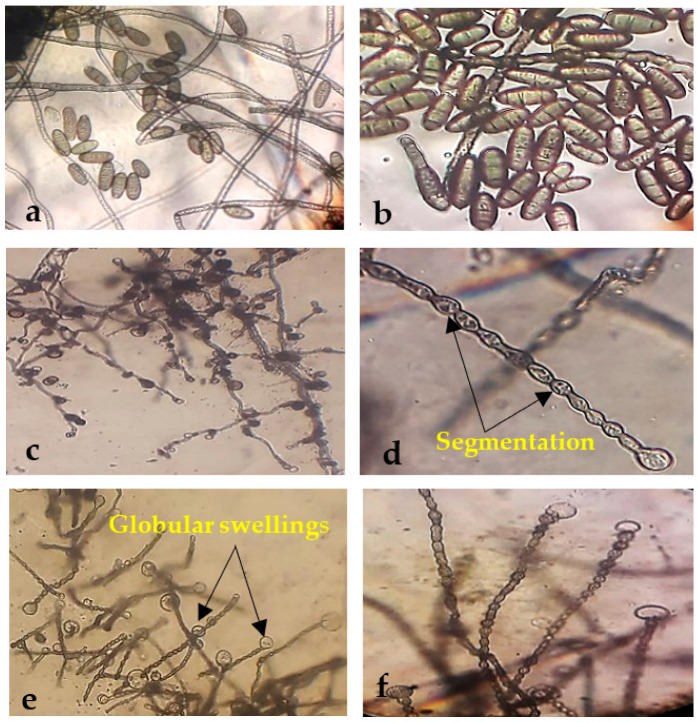
Morphological abnormalities in the mycelia of *Helminthosporium* sp. upon interaction with endophytic bacteria. Images (**a**,**b**) are the untreated (control) *Helminthosporium* sp. mycelia and spores. (**c**,**d**) show the swelling and deformity of *Helminthosporium* sp. mycelia treated with *B. velezensis*. (**e**,**f**) were representatives of swelling, segmentation, and deformation of *Helminthosporium* sp. mycelia treated with *B. amyloliquefaciens*.

The treatment with *B. amyloliquefaciens* and *B. velezensis* caused abnormal mycelial growth and significant morphological changes in *Alternaria* sp. and *Helminthosporium* sp., primarily manifesting as contraction, collapse, deformation, deformity of the conidium, and globular swellings at the tips of hyphal strands (Figure 5 and Figure 6c–f). In contrast, the mycelia of the control group were straight and well developed (Figure 5 and Figure 6a,b).

### 3.4. Bioassay and Biological Activity of the Crude Extracts of Endophytic Bacterial Strains

**Figure 7:**
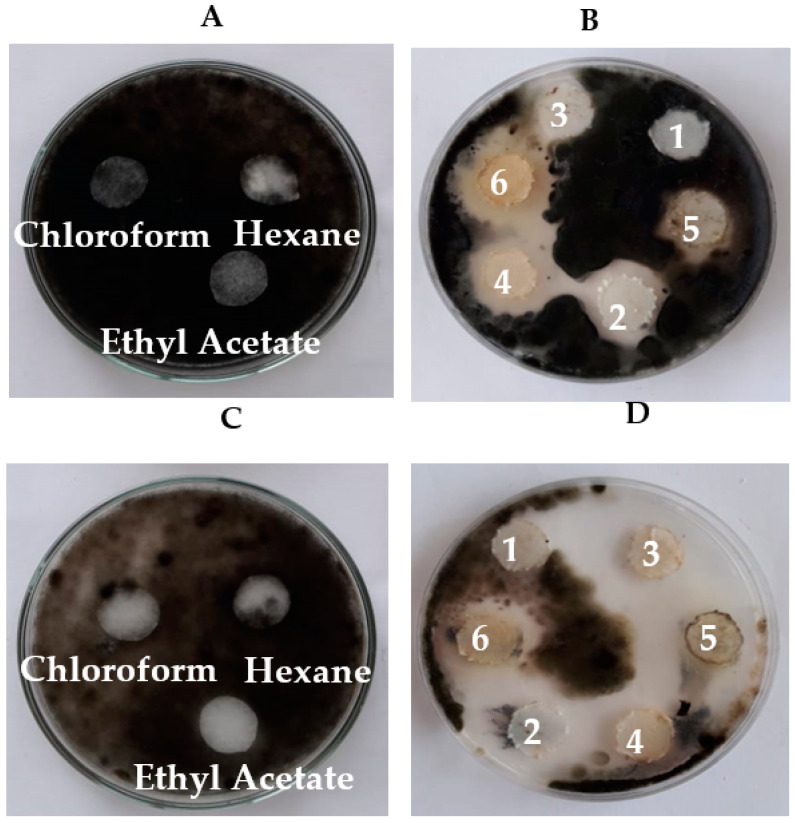
Bioassay activity using the filter paper diffusion method of the antifungal compounds extracted by different types of solvents from *B. amyloliquefaciens and B. velezensis* using *Alternaria* sp. and *Helminthosporium* sp. as test microorganisms. (**A**,**C**) represent the effect of different solvents (50 μL) on *Helminthosporium* sp. and *Alternaria* sp., *respectively* (control). (**B**,**D**) represent the effect of solvent extracts of *B. amyloliquefaciens and B. velezensis* (50 μL) on *Helminthosporium* sp. *and Alternaria* sp., respectively. (**1**) *B. velezensis* methanol Chloroform extract (**2**) *B. amyloliquefaciens* ethyl acetate extract (**3**) *B. velezensis* ethyl acetate extract (**4**) *B. amyloliquefaciens* methanol and chloroform extract. Hexane extracts of *B. velezensis* (**5**) and *B. amyloliquefaciens* (**6**).

The biological activity of the crude extracts of endophytic bacterial strains (*B. amyloliquefaciens* and *B. velezensis*) was investigated using the filter paper diffusion method against both *Alternaria* sp. and *Helminthosporium* sp. Our results showed that all solvents (control) had no inhibitory effects on *Alternaria* sp. and *Helminthosporium* sp. as seen in Figure 7A,C. Figure 7B,D, shows that the solvent extracts of *B. amyloliquefaciens* and *B. velezensis* had inhibitory effects on both fungal pathogens.

### 3.5. Gas Chromatography and Mass Spectrometry (GC–MS)

**Figure 8:**
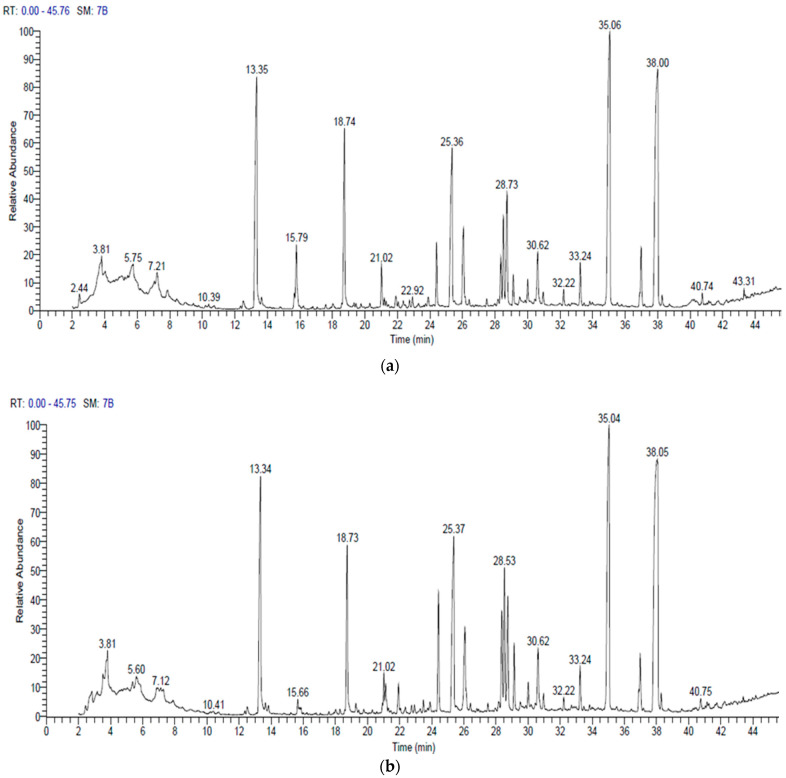
GC-MS chromatogram of bioactive compounds in hexane extracts of endophytic *B. amyloliquefaciens* (**a**) and *B. velezensis* (**b**).

The bioactive components of *B. amyloliquefaciens* and *B. velezensis* were analyzed using GC-MS chromatography (Figure 8 and Table 4 and Table 5). The detected compounds’ names, molecular weights, molecular formulas, retention times, and quantities are listed (Table 4 and Table 5). There were substantial peaks in the cell-free extracts of the two bacterial strains among these bioactive chemicals, implying that they play a significant role in antibacterial and antifungal activity. These chemicals include: Bis (2-ethylhexyl) phthalate, followed by Bis (2-ethylhexyl) ester, *N*,*N*-Dimethyldodecylamine (Tertiary amine), Dibutyl phthalate, Methyl palmitate, and Ethyl hexadecanoate (Figure 8 and Table 4 and Table 5).

### 3.6. In Vivo Evaluation of B. amyloliquefaciens Effects against Alternaria sp. Infected Pepper Plants

**Figure 9:**
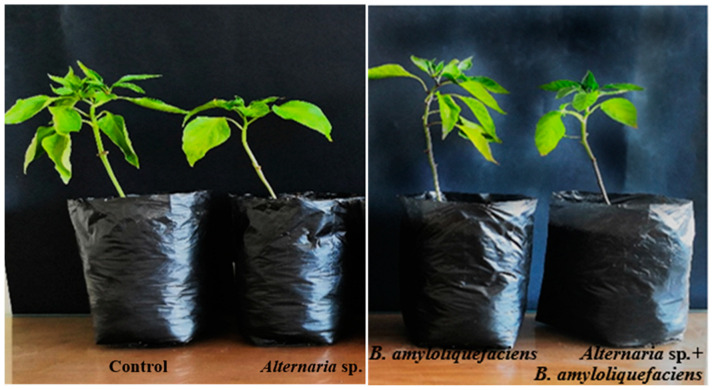
In vivo antagonistic activity of *B. amyloliquefaciens* against *Alternaria* sp. infection of pepper plants.

The most active bacterial endophyte in our study (*B. amyloliquefaciens*) was selected to act as a biocontrol agent via a greenhouse experiment. *Alternaria* sp. was inoculated into pepper plants either in the presence or absence of *B. amyloliquefaciens*. The positive effect of the application of *B. amyloliquefaciens* on pepper TFW, TDW, and plant heights were confirmed (Table 6). The morphological changes between the different treatments are illustrated in Figure 9. Generally, the assessed growth parameters were significantly reduced in pepper plants infected with *Alternaria* sp. compared with the healthy control ones. However, these growth traits significantly increased with *B. amyloliquefaciens*, regardless of whether the plants were infected or not. In non-infected pepper leaves, the application of *B. amyloliquefaciens* significantly improved TFW (8.36 g/plant), TDW (1.4299 g/plant), and plant height (28 cm/plant). Also, Alternaria sp. exhibited the highest DI (80%) in the control plants, while with *B. amyloliquefaciens* inoculation, DI was greatly reduced in Alternaria sp. infected pepper plants (40%), as seen in Table 6. *B. amyloliquefaciens* reduced the disease symptoms; therefore, *B. amyloliquefaciens* exhibited strong antagonism toward *Alternaria* sp. infection and improved the growth of the infected pepper plants.

## 4. Discussion

The increased usage of chemical compounds to maintain healthy crops and high productivity has detrimental consequences for nature, animals, and humans [3,44]. Endophytes have a higher antagonistic potential against plant disease than microorganisms isolated from the rhizosphere or soil because they exist in a stable environment inside the plant [11] and can be found in various host plants [20,22,45]. Endophytes are implicated in the control of plant disease, development of plant tolerance, plant growth promotion, nitrogen fixation, synthesis of novel bioactive compounds, and detoxification of toxic pesticides [46,47]. Moreover, they produce secondary metabolites of biotechnological interest with a pharmaceutical application [48]. Bacterial endophytes vary among organs, tissues, soil, and plants [13].

Some studies consider bacterial endophytes as potential biocontrol agents for various hazardous fungi [6,7]. Selim et al. [17] and Riera et al. [49] revealed that *Streptomyces*, *Pseudomonas*, *Bacillus*, and *Agrobacterium* have long been the most important bacteria genera for the production of active antimicrobial substances. Massawe et al. [23] isolated and characterized *Bacillus* strains with volatile organic compounds (VOCs) acting against *Sclerotinia sclerotiorum*. Earlier investigations documented that antibiotics, such as mycosubtilins, iturins, and bacillomycins, are active metabolites with antimicrobial activities produced by *B. subtilis* [50,51,52].

*Bacillus* spp. can be used to develop effective microbial biopesticides in the form of biological control agents [4]. Olanrewaju et al. [53] reported that *Bacillus* sp. forms beneficial relationships with plants directly or indirectly. *B. velezensis* and *B. amyloliquefaciens* are Gram-positive bacteria that have been used to promote the growth of numerous plants directly or indirectly as they are efficient in plant colonization and commercialized around the world [13,23,54]. The antifungal mechanisms of *B. velezensis* and *B. amyloliquefaciens* are the same, whether through direct antibiosis or plant-mediated induced disease resistance [55,56,57]. As secondary metabolites, *B. velezensis* and *B. amyloliquefaciens* produce several antimicrobial compounds against various phytopathogens [58]. Some fungal pathogens, such as *Helicobasidium purpureum*, *F. oxysporum*, and *Rhizoctonia solani*, are inhibited by the *B. velezensis* strain FKM10. [59,60]. *B. velezensis* can also cause the development of systemic resistance in plants [59]. In some experiments, *B. velezensis* was found to produce several metabolites related to disease resistance, including NH_3_, antimicrobial proteins, polyketides, and siderophores [55,58,60,61]. 

The optical microscopic examination of *Alternaria* sp. and *Helminthosporium* sp. revealed that treatment with *B. amyloliquefaciens* and *B. velezensis* caused abnormal mycelial growth. These anomalies showed a problem with fungal cell wall formation [54]. Zhao et al. [62] noticed abnormal morphological changes in the fungal mycelia of *F. oxysporum*, *Magnaporthe grisea*, and *Alternaria* sp. when interacting with endophytes. The *B. velezensis* strain FKM10 destroyed the cell wall and cell membrane upon interacting with *F. verticillioides* [54]. Furthermore, cyclic lipopeptides produced by *B. velezensis* LM2303 affected the cell membrane permeability of *F. graminearumon* [63]. Moon et al. [64] investigated the potential of *B. velezensis* CE 100 in mitigating phytophthora root rot, which suppressed mycelial growth, causing hyphae to enlarge and distort.

The bioactive metabolites of *B. amyloliquefaciens* and *B. velezensis* were analyzed using GC-MS chromatography. The antifungal actions of these extracts could be related to various chemical classes, including esters, fatty acids, aldehydes, tertiary amines, alkaloids, and ketones. Among these bioactive compounds, the two bacterial strains had significant peaks in cell-free extracts, indicating that they played a substantial role in antibacterial and antifungal activities. These compounds include: Bis (2-ethylhexyl) phthalate followed by Bis (2-ethylhexyl) ester, N, *N*-Dimethyldodecylamine (Tertiary amine), Dibutyl phthalate, Methyl palmitate, and Ethyl hexadecanoate. Phthalates have antimicrobial and antifungal activities [65,66,67,68,69]. Al-Bari et al. [70] reported that the Bis (2-ethylhexyl) antimicrobial activity of phthalate was shown against Gram-positive bacteria and several harmful fungi. Kanjana et al. [71] reported that the Bis (2-ethyl hexyl) phthalate had antifungal, antimicrobial, and antioxidant activities. Furthermore, both bacterial extracts contained dibutyl phthalate, which had antimicrobial activity against unicellular and filamentous fungi [72,73].

The high percentage of Bis (2-ethylhexyl) ester in both bacterial extracts had an antifungal activity. Mohamad et al. [9] suggested that the *Bacillus atrophaeus* strain XEGI50 species was a promising candidate as a biocontrol agent. The GC-MS analysis of cell-free extracts showed that numerous compounds had antimicrobial activity, including Bis (2-ethylhexyl) phthalate and Bis(2-ethylhexyl) ester. The antimicrobial activity of N, *N*-Dimethyldodecylamine, *N*-Methyl-*N*-benzyltetradecanamine, and N, *N*-Dimethyltetradecylamine (tertiary amine) against bacteria, yeasts, fungi, and enveloped viruses has been reported. [74,75]. Massawe et al. [23] reported the biocontrol activity of N, *N*-dimethyl-dodecyl amine against *Sclerotinia sclerotiorum*. The antimicrobial activity of these amine oxides was related to their interaction with biological membranes; the permeability of cellular membranes changed, and membrane-dependent activities were inhibited, resulting in cell death. Furthermore, the amine oxides caused K^+^ leakage from cells and lysis of osmotically stabilized protoplasts, which inhibited glycolysis [74,75].

Methyl palmitate (fatty acid methyl esters) detected in both bacterial extracts exhibited antibacterial and antifungal activities, damaging microbial cellular membranes [76]. Chandrasekaran et al. [77] argued that fatty acid methyl ester extract showed moderate antifungal activity against two *Aspergillus* spp. Moreover, ethyl hexadecanoate had antimicrobial, antioxidant, and pesticidal activities [78]. The least observed in either of the bacterial extracts were octyl hexadecanoate, diethyl phthalate, 2-phenyltridecane, methyl 10-methyl undecanoate, 5-octadecene, octadecane, and methyl tetradecanoate that had antimicrobial, antioxidant, and anticancer activities [71,78,79].

Some *Bacillus* strains produce volatile organic compounds (VOCs) that may act as antifungal agents against various soil-borne diseases and limit fungal growth [23,48]. These VOCs can diffuse among soil particles and spread far from where they were applied, inhibiting pathogens without coming into direct contact with them [80,81]. For example, the VOCs produced by *B. amyloliquefaciens* may inhibit *F. oxysporum* mycelial development and spore germination [21,82]. Gao et al. [20] and Jiang et al. [83] reported that different strains of *B. velezensis* suppressed the growth of *B. cinerea* by different numbers of VOCs. Also, Reda et al. [84] indicated that the *B. amyloliquefaciens* S_5_I_4_ strain produced bioactive antimicrobial compounds.

The enhancing capacity of *B. amyloliquefaciens* for pepper plant growth is in coherence with Shahzad et al. [85] and Rashad et al. [19], who documented that *B. amyloliquefaciens* RWL-1 and GGA inoculation significantly enhanced the growth traits of tomato and garlic plants under both diseased and non-diseased conditions of *F. oxysporum* and *S. cepivorum*. The extensively distinguished mechanisms for plant growth promotion caused by *B. amyloliquefaciens* are through phytohormones, providing the essential nutrients, N_2_ fixation, and phosphate solubilization [18]. In addition, endophytic bacteria may promote plant growth through biofertilization [19]. Shahzad et al. [85] discovered that the endophytes’ capability to produce secondary metabolites provided additional support to plants and increased plant development, increasing their resilience to biotic and abiotic challenges. These mechanisms can contribute to the plant growth-promoting potential of *B. amyloliquefaciens* on pepper plants.

## 5. Conclusions

Controlling plant pathogen diseases in a safe, effective, and alternative manner has become increasingly crucial for improving the quality of agricultural products. Compared to chemical control, biological control using antagonistic microorganisms, such as bacteria, is a long-term approach to inhibiting plant pathogens. The novelty of this work is the isolation of new local strains of endophytic bacteria and the production of several antimicrobial metabolites associated with the biocontrol of *Alternaria*, which can cause serious diseases to important vegetable crops in Egypt. *Bacillus* species, as biocontrol agents, could inhibit potential plant pathogens. The *B. amyloliquefaciens* MZ945930 and *B. velezensis* MZ945929 strains in this study, shared the same antifungal mechanisms by direct antibiosis against *Alternaria* sp. and *Helminthosporium* sp. Moreover, suppressive effects were associated with a variety of secondary metabolite secretions. The resulting bacterial crude extracts from both bacterial strains were promising as they have shown the highest antifungal activities. Also, the in vivo results emphasized the significance of the effect of *B. amyloliquefaciens* on pepper growth under both the control and diseased conditions caused by *Alternaria* sp. Therefore, the present study encourages the use of these bacterial strains as biocontrol agents in agriculture.

## Figures and Tables

**Table 1:** Endophytic bacterial isolates from various crop plants were isolated and tested for inhibitory activity against *Alternaria* sp. and *Helminthosporium* sp. using a dual culture technique.

Plants	Organ	Isolate No.	Antagonistic Activity	
			*Alternaria* sp.	*Helminthosporium* sp.
*Solanum melongena*	Leaf, stem and Root	E_1_	+	+
		E_2_	−	+
		E_3_	+++	+
		E_4_	+	+
		E_5_	+	++
*Allium cepa*	Root and leaf	O_1_	+	+
		O_2_	+	+
*Portulaca oleracea*	leaf	R_1_	++	++
		R_2_	++	++
		R_3_	−	−
		R_4_	+	+
		R_5_	++	+
		R_6_	−	−
		R_7_	+	++
*Coriandrum sativum*	leaf	K_1_	+	+
*Pisum sativum*	Leaf and stem	P_1_	+	+
		P_2_	+	+
		P_3_	+	+
		P_4_	+	+
		P_5_	+	+
*Solanum lycopersicum*	leaf	T_1_	+	+
		T_2_	+	+
		T_4_	+	++
		T_5_	+++	+++
*Brassica oleracea*	leaf	C_1_	++	+++

Data are based on four replicates of each experiment. + represents 2–5 mm wide zone; ++ represents 5–10 mm wide zone and +++ represents > 10 mm wide zone.

**Table 2:** Antagonistic activity of selected endophytic bacterial isolates against *Helminthosporium* sp. and *Alternaria* sp. in vitro.

Isolate Number	Inhibition of Mycelial Growth (cm)			
	*Alternaria* sp.		*Helminthosporium* sp.	
	Bacterial Filtrate (Cell-Free Culture) (150 μL)	Bacterial Disc (15 mm)	Bacterial Filtrate (Cell-Free Culture) (150 μL)	Bacterial Disc (15 mm)
E_3_	3.8 ± 0.201 ^a^	2.6 ± 0.137 ^c^	2.5 ± 0.132 ^a^	2.5 ± 0.132 ^cd^
E_5_	3 ± 0.159 ^b^	2.7 ± 0.143 ^bc^	2.7 ± 0.143 ^a^	2.37 ± 0.125 ^cd^
R_1_	3.6 ± 0.191 ^a^	3.1 ± 0.164 ^b^	2.7 ± 0.143 ^a^	2.25 ± 0.119 ^d^
R_2_	4 ± 0.212 ^a^	3 ± 0.158 ^bc^	2.5 ± 0.132 ^a^	2.8 ± 0.148 ^c^
T_4_	3.8 ± 0.201 ^a^	2.1 ± 0.111 ^d^	1.8 ± 0.095 ^b^	0.9 ± 0.064 ^e^
T_5_	3.6 ± 0.191 ^a^	4.0 ± 0.212 ^a^	2.6 ± 0.138 ^a^	3.9 ± 0.329 ^b^
C_1_	4.2 ± 0.222 ^a^	3.1 ± 0.164 ^b^	2.8 ± 0.148 ^a^	4.77 ± 0.252 ^a^

E_3_, E_5_, R_1_, R_2_, T_4_, T_5_, and C_1_ are selected endophytic bacterial cultures isolated from different plant species. Data are the mean of 3 replicates ± standard error. Different letters in the same column denote significant difference at the *p* < 0.05 level by Duncan’s new multiple range test.

**Table 3:** Morphological and biochemical tests of as *Bacillus amyloliquefaciens* and *Bacillus velezensis*.

Characteristics	*B. velezensis*	*B. amyloliquefaciens*
**Shape**	Rod	Rod
**Gram stain**	+	+
**Spore formation**	+	+
**Oxidase**	+	+
**Indole test**	-	-
**Hydrogen sulphide**	-	-
**Catalase reaction**	+	+
**Methyl red test**	-	-
**Nitrate (reduction)**	+	+
**Voges-proskauer test**	+	+
**Urease**	-	-
**Hydrolyzed Starch**	+	+
**Gelatin liquefaction**	+	+
**Growth in 10** **% NaCl**	-	+

**Table 4:** List of identified bioactive compounds of endophytic *B. amyloliquefaciens* extract through GC-MS analysis.

No.	Compound Name and Class	Molecular Formula	MW	Area%	RT (min)	Base Peak (100%)
1	6,6-Dimethyl-1,3-heptadien-5-ol (Alchols)	C_9_H_16_O	140	0.46	2.44	57.0
2	Benzaldehyde, 3-benzyloxy-2-fluoro-4-methoxy benzaldehyde (Aldehyde)	C_15_H_13_FO_3_	260	2.91	3.69	91.0
3	Chloromethyl benzene (Halobenzene)	C_7_H_7_Cl	126	0.73	3.80	91.0
4	decyloxy anime (Amines)	C_10_H_23_NO	173	0.98	5.75	43.0
5	Naphthalene, 1,2,3,4-tetrahydro-5-methyl-(poly nuclear aromatic cpds)	C_11_H_14_	146	0.61	7.22	131.0
6	1,3,5-Triazine-2,4-diamine, 6-chloro-*N*-ethyl- (Heterocyclic cpds)	C_5_H_8_ClN_5_	173	0.34	7.84	43.0
7	*N*,*N*-Dimethyldodecylamine (Tertiary amine)	CH_3_(CH_2_)_11_N(CH_3_)_2_	213	11.84	13.34	58.0
8	5-Octadecene (Alkene)	C_18_H_36_	252	0.21	15.65	55.0
9	Cetene (Alkene)	C_16_H_32_	224	0.21	15.65	41.0
10	Diethyl phthalate (Esters)	C_12_H_14_O_4_	222	2.05	15.79	149.0
11	*N*,*N*-Dimethyltetradecylamine (Tertiary amine)	C_16_H_35_N	241	6.57	18.74	58.0
12	1-Docosene (Alkene)	C_22_H_44_	308	1.38	21.02	55.0
13	9-Nonadecene (Alkene)	C_19_H_38_	266	1.38	21.02	41.0
14	9-Eicosene, (E)- (Alkene)	C_20_H_40_	280	1.38	21.02	57.0
15	Octadecane (Alkane)	CH_3_(CH_2_)_16_CH_3_	254	0.19	21.19	57.0
16	4-Phenyleicosane (Alkyl benzene)	C_26_H_46_	358	0.28	22.36	91.0
17	Methyl palmitate (fatty ester)	C_17_H_34_O_2_	270	2.11	24.40	74.0
18	Dibutyl phthalate (Esters)	C_16_H_22_O_4_	278	8.10	25.35	149.0
19	Ethyl hexadecanoate (fatty esters)	C_18_H_36_O_2_	284	3.25	26.06	88.0
20	Methyl linoleate (un-saturated fatty ester)	C_19_H_34_O_2_	294	1.47	28.35	67.0
21	Methyl 11-Octadecenoate (unsaturated fatty ester)	C_19_H36O2	296	2.86	28.51	55.0
22	3-(*N*-Benzyl-*N*-methylamino)-1,2-propanediol (Amino alchol)	C_11_H_17_NO_2_	195	4.09	28.73	91.0
23	Methyl stearate (Sat. fatty acids)	C_19_H_38_O_2_	298	0.94	29.12	74.0
24	1,3,5(10)-Oestratrien-17α-ol (Chlosterol)	C_18_H_24_O	256	0.36	29.52	43.0
25	Ethyl oleate (un-sat. fatty acids)	C_20_H_38_O_2_	310	0.74	30.01	55.0
26	Ethyl Octadecanoate (Sat.fatty esters)	C_20_H_40_O_2_	312	1.91	30.62	88.0
27	*N*-Methyl-*N*-benzyltetradecanamine (Tertiary amine)	C_22_H_39_N	317	1.50	33.24	134.0
28	1-Phenylacetone (ketone)	C_9_H_10_O_2_	134	1.50	33.24	43.00
29	Bis (2-ethylhexyl) ester (Esters)	C_22_H_42_O_4_	370	18.59	35.06	129.0
30	Octyl hexadecanoate (Sat. fatty acids)	C_24_H_48_O_2_	368	2.33	36.99	257.0
31	Bis(2-ethylhexyl) phthalate (Esters)	C_24_H_38_O_4_	390	20.36	38.00	149.0

RT: Retention time; MW: Molecular weight.

**Table 5:** List of identified bioactive compounds of endophytic *B. velezensis* extract by GC-MS analysis.

No.	Compound Name and Class	Molecular Formula	MW	Area %	RT (min)	Base Peak (100%)
1	4-isopropenyl-1-methylcyclohexene (R(+) Limonene)	C_10_H_16_	136	1.42	2.84	68.0
2	Perilla alcohol (Alkaloids)	C_10_H_16_O	152	0.40	3.17	41.0
3	Benzaldehyde, 3-benzyloxy-2-fluoro-4-methoxy- (Aldehyde)	C_15_H_13_FO_3_	260	0.59	3.53	91.0
4	Chloromethyl benzene (Halobenzene)	C_7_H_7_Cl	126	0.32	3.81	91.0
5	Dodec-1-ene (Alkane)	C_12_H_24_	168	0.25	5.38	43.0
6	5-Isopropenyl-2-methyl-2-cyclohexen-1-one (alkaloids)	C_10_H_16_O	150	0.36	6.86	82.0
7	1,3,5-Triazine-2,4-diamine, 6-chloro-*N*-ethyl-(Heterocyclic cpds)	C_5_H_8_ClN_5_	173	0.70	7.29	43.0
8	4,4,6-Trimethyl-6-phenyl-1,3-oxazinane-2-thione (Heterocycliccpds)	C_13_H_17_NOS	235	0.34	7.29	118.0
9	*N*,*N*-Dimethyldodecylamine (Tertiary amine)	CH_3_(CH_2_)_11_N(CH_3_)_2_	213	9.08	13.34	58.0
10	Methyl 10-methylundecanoate (saturated Fatty ester)	C_13_H_26_O_2_	214	0.17	13.83	74.0
11	Diethyl phthalate (Esters)	C_12_H_14_O_4_	222	0.17	15.77	149.0
12	Methyl tetradecanoate (Sat. fatty acids)	C_15_H_30_O_2_	242	0.29	19.27	74.0
13	*N*,*N*-Dimethyltetradecylamine (Tertiary amine)	C_16_H_35_N	241	5.21	18.73	58.0
14	Methyl 12-methyltetradecanoate (Sat. fatty acids)	C_16_H_32_O_2_	256	1.10	20.92	74.0
15	9-Eicosene, (E)-(Alkene)	C_20_H_40_	280	1.06	21.02	57.0
16	Methyl 9-oxodecanoate (Esters)	C_11_H_20_O_3_	200	0.87	21.93	43.0
17	Methyl 14-methylpentadecanoate (Sat. fatty esters)	C_17_H_34_O_2_	270	3.88	23.48	74.0
18	Dibutyl phthalate (Esters)	C_16_H_22_O_4_	278	8.18	25.37	149.0
19	Ethyl hexadecanoate (Fatty esters)	C_18_H_36_O_2_	284	3.24	26.06	88.0
20	Methyl linoleate (un-saturated fatty ester)	C_19_H_34_O_2_	294	2.85	28.36	67.0
21	Methyl elaidate (un-saturated fatty ester)	C_19_H_36_O_2_	296	4.03	28.53	55.0
22	Methyl stearate (Sat. fatty acids)	C_19_H_38_O_2_	298	1.87	29.13	74.0
23	Ethyl 9-octadecenoate (unsat. fatty ester)	C_20_H_38_O_2_	310	0.68	30.01	55.0
24	Ethyl Octadecenoate (Sat. fatty ester)	C_20_H_20_O_2_	312	1.88	30.62	88.0
25	*N*-Methyl-*N*-benzyltetradecanamine (Tertiary amine)	C_22_H_39_N	317	1.30	33.24	134.0
26	1-Phenyl acetone (ketone)	C_9_H_10_O	134	1.30	33.24	43.0
27	Bis (2-ethylhexyl) ester (Esters)	C_22_H_42_O_4_	370	14.98	35.04	129.0
28	Octyl hexadecanoate (Sat. fatty acids)	C_24_H_48_O_2_	368	2.15	36.98	257.0
29	Bis(2-ethylhexyl) phthalate (Esters)	C_24_H_38_O_4_	390	24.39	38.04	149.0
30	Octadecanoic acid (Sat. fatty acids)	C_18_H_36_O_2_	284	0.36	40.75	43.0
31	Dinonyl phthalate (Esters)	C_26_H_42_O_4_	418	0.17	41.14	149.0

RT: Retention time; MW: Molecular weight.

**Table 6:** The effect of *B. amyloliquefaciens* on pepper growth promoting traits and disease incidence (DI) (%) during *Alternaria* sp. invasion.

Treatments	Plant Height (cm/Plant)	TFW (g/Plant)	TDW (g/Plant)	Disease Incidence DI (%)
Control	26 ± 0.687 ^ab^	7.73 ± 0.205 ^b^	1.339 ± 0.035 ^a^	0 ± 0.0 ^c^
*Alternaria* sp.	20.8 ± 0.55 ^c^	3.81 ± 0.101 ^d^	0.668 ± 0.018 ^c^	80 ± 2.07 ^a^
*B. amyloliquefaciens*	28 ± 0.74 ^a^	8.36 ± 0.221 ^a^	1.4299 ± 0.038 ^a^	0 ± 0.0 ^c^
*Alternaria* sp. *+ B. amyloliquefaciens*	24.5 ± 0.648 ^b^	5.88 ± 0.156 ^c^	0.8024 ± 0.021 ^b^	40 ± 1.096 ^b^

The values are the means of 10 replicates ± standard error (*n* = 10). The same letter within each column indicates no significant difference between the treatments (*p* ≤ 0.05) as determined by Duncan’s multiple range test.

## Data Availability

The relevant datasets supporting the results of this article are included within the article and the [GenBank NCBI] at: https://www.ncbi.nlm.nih.gov/nuccore/MZ945930.1/ (accessed on 1 September 2021).
